# Association Between Neighborhood-Level Social Determinants of Health and Access to Pediatric Appendicitis Care

**DOI:** 10.1001/jamanetworkopen.2021.48865

**Published:** 2022-02-16

**Authors:** Megan E. Bouchard, Kristin Kan, Yao Tian, Mia Casale, Tracie Smith, Christopher De Boer, Samuel Linton, Fizan Abdullah, Hassan M. K. Ghomrawi

**Affiliations:** 1 Division of Pediatric Surgery, Department of Surgery, Ann & Robert H. Lurie Children’s Hospital of Chicago, Chicago, Illinois; 2 Division of Advanced General Pediatrics and Primary Care, Department of Pediatrics, Ann & Robert H. Lurie Children’s Hospital of Chicago, Chicago, Illinois; 3 Departments of Surgery and Pediatrics, Northwestern University Feinberg School of Medicine, Chicago, Illinois; 4 Population Health Analytics, Division of Data Analytics and Reporting, Ann & Robert H. Lurie Children’s Hospital of Chicago, Chicago, Illinois

## Abstract

**Importance:**

Presenting with complicated appendicitis, which is associated with higher rates of complications and readmissions compared with simple appendicitis, may indicate delayed access to care. Although both patient-level and neighborhood-level social determinants of health are associated with access to care, little is known about the association between neighborhood factors and access to acute pediatric surgical care.

**Objective:**

To examine the association between neighborhood factors and the odds of presenting with complicated appendicitis and unplanned postdischarge health care use.

**Design, Setting, and Participants:**

A retrospective cohort study of patients aged 18 years or younger diagnosed with appendicitis was conducted. Discharge data from October 1, 2015, to September 30, 2018, were obtained from the Pediatric Health Information System Database and linked to the Child Opportunity Index (COI) 2.0 Database. Data analysis was conducted from January 1 through July 1, 2021.

**Exposures:**

The COI, a composite score of zip code neighborhood opportunity level information, divided into quintiles ranging from very low to very high opportunity.

**Main Outcomes and Measures:**

Based on COI level, the main outcome was the odds of presenting with complicated appendicitis, which was defined using the Agency for Healthcare Research and Quality–specified *International Statistical Classification of Diseases, 10th Edition*, *Clinical Modification* codes. The secondary outcome was the odds of unplanned postdischarge health care use (emergency department visits and/or readmissions) for patients with simple and with complicated appendicitis.

**Results:**

A total of 67 489 patients (mean [SD] age, 10.5 [3.9] years) had appendicitis, with 31 223 cases (46.3%) being complicated. A total of 1699 patients (2.5%) were Asian, 24 234 (35.9%) were Hispanic, 4447 (6.6%) were non-Hispanic Black, and 29 234 (43.3%) were non-Hispanic White; 40 549 patients (60.1%) were male; and 32 343 (47.9%) were publicly insured. Patients living in very low–COI neighborhoods had 28% higher odds of presenting with complicated appendicitis (odds ratio, 1.28; 95% CI, 1.20-1.35) compared with those in very high–COI neighborhoods. There was no significant association between COI level and unplanned postdischarge health care use (very high COI, 20.8%; very low COI, 19.1%).

**Conclusions and Relevance:**

In this cohort study, children from lower-COI neighborhoods had increased odds of presenting with complicated appendicitis compared with those from higher-COI neighborhoods, even after controlling for patient-level social determinants of health factors. These findings may inform policies and programs that seek to improve access to pediatric surgical care.

## Key Points

**Question:**

Using appendicitis as a case study, are neighborhood-level social determinants of health associated with access to pediatric surgical care?

**Findings:**

In this cohort study including 67 489 children, after controlling for patient-level social determinants of health, children from lower-opportunity neighborhoods had 5% to 28% increased odds of presenting with complicated appendicitis, an indicator of delayed access, compared with those from higher-opportunity neighborhoods. There was no significant difference in unplanned postdischarge health care use based on neighborhood opportunity level.

**Meaning:**

The findings of this cohort study may inform policies and programs that seek to improve access and disparities in pediatric surgical care.

## Teaser

This cohort study examines the incidence of complicated appendicitis in children from neighborhoods with differing indicators of a child’s health and development.

## Introduction

Access to pediatric surgical care is important because delay in diagnosis and care can lead to increased severity of disease at the time of presentation, higher risk of emergency surgery, need to return to the operating room, admission to the intensive care unit, long length of stay, high health care costs, and postoperative complications.^1^ Access to surgical care is affected by many factors, including both patient-level and neighborhood-level social determinants of health (SDOH) factors.^2,3^ Although a large body of literature exists on the association between patient-level SDOH and access to and outcomes of surgical care, little is known about neighborhood SDOH, particularly in pediatric surgery.^4^ The few studies that evaluated the association of neighborhood SDOH with surgical access and outcomes either were limited to adults or evaluated only 1 or 2 neighborhood characteristics, such as zip code–level median household income.^5,6,7^ Meanwhile, in nonsurgical pediatric studies, neighborhood SDOH was also associated with health outcomes, such as differences in rates of hospitalization for asthma, firearm injuries, and acute care visits.^8,9^

Appendicitis is the most common acute pediatric surgical condition, with 80 000 cases in the US annually, and accounts for approximately 20% of all inpatient pediatric surgical care.^10,11^ Appendicitis has 2 defined disease stages: simple and complicated. Approximately 30% of pediatric patients with appendicitis present with complicated appendicitis, defined as perforation or gangrenous changes of the appendix that may be accompanied by intraabdominal abscess or phlegmon.^11,12^ Complicated appendicitis is associated with higher surgical complication rates, longer lengths of stay, greater costs, and higher readmission rates compared with simple appendicitis.^4,13^ Presenting with complicated appendicitis has been used as an indicator for potentially delayed access to definitive diagnosis and care.^10,11^ For example, the incidence of complicated appendicitis is highest among patients who are publicly insured, low income, of an ethnic or racial minority group, or living in rural communities.^14,15,16^ Thus, appendicitis serves as a useful case study to evaluate the association between neighborhood SDOH and access to pediatric surgical care.

With increased interest from health care systems, public health organizations, and third-party payers in addressing and alleviating the adverse outcomes associated with neighborhood SDOH in patient access and outcomes,^17,18,19,20^ we sought to examine the association between presenting with complicated appendicitis and neighborhood SDOH, as measured by the Child Opportunity Index (COI), a comprehensive measure of 29 neighborhood SDOH indicators known to affect children’s health outcomes.^21^ In addition, we evaluated whether COI is associated with differences in unplanned postdischarge health care use, including emergency department visits and readmissions. We hypothesized that children from lower-COI neighborhoods would have increased odds of presenting with complicated appendicitis and unplanned health care use.

## Methods

### Study Design and Data Sources

A retrospective cohort study of pediatric appendicitis outcomes was conducted using the Pediatric Health Information System (PHIS) database, a national administrative database for inpatient, ambulatory surgery, emergency department, and observation unit visits at 49 tertiary pediatric centers. Pediatric Health Information System data points are entered by individual hospitals; however, PHIS maintains an oversight program to ensure high data quality and integrity. The PHIS has been used extensively to study pediatric patient outcomes.^22,23^ With an estimated 25 600 appendectomies performed annually at PHIS hospitals, the PHIS database captures approximately one-third of the patients with a diagnosis of appendicitis.^24^ In addition, unlike other databases, the PHIS has the advantage of including both observation and inpatient cases, which is particularly important to capture patients with simple (primarily observation) and complicated (primarily inpatient) appendicitis.^25^ This study was approved by the Ann & Robert H. Lurie Children’s Hospital of Chicago Institutional Review Board, which waived participant consent because data were deidentified. This study followed the Strengthening the Reporting of Observational Studies in Epidemiology (STROBE) reporting guideline for cohort studies.

We linked PHIS to the Child Opportunity Index (COI) 2.0 database at the zip code level. The COI captures zip code neighborhood opportunity level information and is available for nearly all US Census tract neighborhoods using 2015 data compiled from public sources, including the Census Bureau, National Center for Health Statistics, Department of Education, and Environmental Protection Agency.^21^ The COI database compiles 29 independent indicators of neighborhood characteristics that are known to influence a child’s health and development, including but not limited to access to and quality of education, health care, green space, nutritious food, toxin-free environments, safety, and socioeconomic resources. The COI indicators are further subdivided into 3 themed subdomains: education, health/environment, and social/economic (eAppendix in the Supplement). The overall COI and subdomain scores are calculated and divided into nationally normalized quintiles, with scores from 1 (very low) to 100 (very high) opportunity levels. The contribution of each indicator to the overall score is weighted based on how strongly an indicator estimates the long-term health outcomes. The COI has been used to increase equity awareness, evaluate how neighborhoods affect child health, and inform resource allocation, public health interventions, and policy development.^21,26^ Zip code COI levels were cross-linked to zip codes included in the PHIS database.

### Study Population

Patients aged 18 years or younger who were diagnosed with appendicitis (*International Statistical Classification of Diseases, Tenth Revision, Clinical Modification* [*ICD-10-CM*] codes K35.2, K35.3, K35.80, K35.89, and K37; Agency for Healthcare Research and Quality 2018) between October 1, 2015, and September 30, 2018, were identified in the PHIS (N = 77 073) (eTable 1 in the Supplement).^27^ This timeline was chosen to be contemporaneous with the COI data and to ensure consistent *ICD-10*-*CM* coding across the study period, since the diagnosis codes for appendicitis were modified on October 1, 2018. Patients were included if their encounter status was observation, emergency, or inpatient; however, to avoid the inclusion of interval appendectomies, patients with elective encounters were excluded (n = 7816). In addition, patients listed as newborn (n = 4) or trauma (n = 12), had a missing COI variable (n = 248), or had 2 distinct discharge identification numbers on the same day from the same hospital (n = 1504) were excluded.

### Outcomes and Covariates

The primary outcome was presenting with complicated appendicitis. The Agency for Healthcare Research and Quality specification (*ICD-10-CM* codes K35.2 and K35.3) was used to define complicated appendicitis in the main analysis of this study.^27,28^

The secondary outcome was having a postdischarge emergency department visit and/or unplanned 30-day readmission for patients with simple and with complicated appendicitis; we stratified by subtype of appendicitis because patients with complicated appendicitis are expected to have higher rates of complications and thus are more likely to present for postdischarge health care use.

Based on previous literature on factors associated with appendicitis outcomes, models were adjusted for age 18 years or younger, sex, race and ethnicity as reported in the PHIS database (Asian, Hispanic, non-Hispanic Black, non-Hispanic White, and other [Native Hawaiian/Pacific Islander, American Indian, and Alaskan Native]), and insurance type (private, public [Medicaid/Children’s Health Insurance Program], other).^4,16^ Age was made a categorical variable given that each age subgroup has different reported rates of complicated appendicitis. Race and ethnicity data were captured from the PHIS database, which receives data from each participating hospital; however, it is unknown how each hospital classifies race and ethnicity and whether the category is self-reported. The PHIS marker for the presence of complex chronic conditions was also included because patients with multiple comorbidities are more likely to have a delay in diagnosis and present with complicated appendicitis.^29^

### Statistical Analysis

Data analysis was conducted from January 1 through July 1, 2021. Descriptive statistics using the χ^2^ test or Wilcoxon rank-sum test are reported to compare patient characteristics between those with simple and with complicated appendicitis. We then used hierarchical logistic regression to assess the odds of presenting with complicated appendicitis as a function of COI and a random intercept for each hospital to control for patient clustering within a hospital. Covariates included age, sex, race, ethnicity, insurance type, and the presence of complex chronic conditions. We estimated a parsimonious model consisting of COI and covariates, as well as additional models that included interaction terms between COI and covariates. The interaction terms were not statistically significant and therefore were excluded from the final model. The choice of the parsimonious model was additionally supported by bayesian information criterion values and other model selection criteria. The same modeling strategy was also performed to examine the association of COI with unplanned health care use (ie, emergency department visit or 30-day readmission).

### Sensitivity Analysis

Recent evidence has shown that the *ICD-10-CM* code K35.3 is not specific and may thus overestimate the number of complicated appendicitis cases in our cohort.^27,28^ To solve this low-specificity concern, we performed a sensitivity analysis defining complicated appendicitis cases using only the *ICD-10-CM* code K35.2, which is highly specific for identifying complicated appendicitis.^30^ An α level of .05 was considered statistically significant. All analyses were performed using SAS software, version 9.4 (SAS Institute Inc).

## Results

A total of 67 489 children with appendicitis were identified; 31 223 children (46.3%) presented with complicated appendicitis (Table 1). The population comprised 40 549 boys (60.1%) and 26 929 girls (39.9%). Race and ethnicity groups were Asian (1699 [2.5%]), Hispanic (24 234 [35.9%]), non-Hispanic Black (4447 [6.6%]), non-Hispanic White (29 234 [43.3%]), and other (Native Hawaiian/Pacific Islander, American Indian, and Alaskan Native; 7875 [11.7%]). Rural residence was reported for x (8.2%) of children with simple appendicitis and x (10.3%) of those with complicated appendicitis. Most children were publicly insured (32 343 [47.9%]) and did not have a complex chronic condition (63 882 [94.7%]). The median age for all children with appendicitis cases and for simple cases was 11 (IQR, 8-14) years, and the median age for those with complicated appendicitis was 10 (IQR, 7-13) years. The proportions of overall appendicitis cases were fairly evenly distributed across COI levels (from 17.0% to 23.0%), and most patients did not visit the emergency department or require readmission within 30 days from discharge (57 131 [84.7%]). In bivariate analysis, compared with patients who had simple appendicitis, there was a greater proportion of children with complicated appendicitis who were Hispanic (12 226 [39.2%] vs 12 008 [33.1%]; *P* < .001), and publicly insured (15 996 [51.2%] vs 16 347 [45.1%]; *P* < .001), presented to the emergency department or were readmitted within 30 days postoperatively (6004 [19.2%] vs 4354 [12.0%]; *P* < .001), or were from a very low–COI neighborhood (8055 [25.8%] vs 7433 [20.5%]; *P* < .001).

**Table 1.** Characteristics of Patients With Appendicitis

Characteristic	Total No.	Appendicitis group, No. (%)		*P* value
		Simple	Complicated	
No.	67 489	36 266 (53.7)	31 223 (46.3)	
Age, y				<.001
Mean (SD)	10.5 (3.9)	11.0 (3.6)	9.9 (4.0)	
Median (IQR) [range]	11 (8-14) [0-18]	11 (8-14) [0-14]	10 (7-13) [0-18]	
Sex				
Female	26 929 (39.9)	14 210 (39.2)	12 719 (40.7)	<.001
Male	40 549 (60.1)	22 049 (60.8)	18 500 (59.3)	
Unknown	11 (0.02)	7 (0.01)	4 (0.01)	
Race and ethnicity				
Asian	1699 (2.5)	869 (2.4)	830 (2.7)	<.001
Hispanic	24 234 (35.9)	12 008 (33.1)	12 226 (39.2)	
Non-Hispanic				
Black	4447 (6.6)	2363 (6.5)	2084 (6.7)	
White	29 234 (43.3)	16 721 (46.1)	12 513 (40.1)	
Other^a^	7875 (11.7)	4305 (11.9)	3570 (11.4)	
Insurance				
Private	28 991 (43.0)	16 776 (46.3)	12 215 (39.1)	<.001
Public (Medicaid/CHIP)	32 343 (47.9)	16 347 (45.1)	15 996 (51.2)	
Other	6155 (9.1)	3143 (8.7)	3012 (9.6)	
Complex chronic condition				
Yes	3607 (5.3)	1877 (5.2)	1730 (5.5)	.04
No	63 882 (94.7)	34 389 (94.8)	29 493 (94.5)	
COI level				
Very low	15 488 (23.0)	7433 (20.5)	8055 (25.8)	<.001
Low	13 535 (20.1)	6897 (19.0)	6638 (21.3)	
Moderate	12 668 (18.8)	6821 (18.8)	5847 (18.7)	
High	11 487 (17.0)	6633 (18.3)	4854 (15.5)	
Very high	14 311 (21.2)	8482 (23.4)	5829 (18.7)	
ED visit/30-d readmission				
Yes	10 358 (15.4)	4354 (12.0)	6004 (19.2)	<.001
No	57 131 (84.7)	31 912 (88.0)	25 219 (80.8)	

Abbreviations: CHIP, Children’s Health Insurance Program; COI, Child Opportunity Index; ED, emergency department.

^a^ Includes Native Hawaiian/Pacific Islander, American Indian, and Alaskan Native.

Full regression results are presented in Table 2. After adjusting for covariates, in comparison with very high COI, the odds ratios of presenting with complicated appendicitis were very low, 1.28 (95% CI, 1.20-1.35); low, 1.20 (95% CI, 1.13-1.27); moderate, 1.16 (95% CI, 1.09-1.22); and high, 1.05 (95% CI, 1.01-1.11) (Table 2). The same pattern of increasing odds of complicated appendicitis with each lower level of COI was also noted within each COI subdomain (educational level, health/environment, and socioeconomic status) (Table 3).

**Table 2.** Association of Overall COI Level and Other Covariates With Likelihood of Presenting With Complicated Appendicitis

Covariate	Adjusted OR (95% CI) of complicated appendicitis
Sex	
Female	1.06 (1.02-1.09)
Unknown	1.07 (0.31-3.70)
Male	1 [Reference]
Age, y	
0-4	4.20 (3.89-4.53)
5-9	1.51 (1.44-1.59)
10-14	1.26 (1.20-1.32)
≥15	1 [Reference]
Race and ethnicity	
Asian	1.31 (1.18-1.46)
Hispanic	1.14 (1.08-1.19)
Non-Hispanic, Black	1.09 (1.02-1.17)
Other^a^	1.05 (0.98-1.11)
Non-Hispanic, White	1 [Reference]
Insurance	
Public (Medicaid/CHIP)	1.09 (1.05-1.14)
Other	1.09 (1.02-1.16)
Private	1 [Reference]
Complex chronic condition	
Yes	1.07 (0.99-1.15)
No	1 [Reference]
Overall COI level	
Very low	1.28 (1.20-1.35)
Low	1.20 (1.13-1.27)
Moderate	1.16 (1.09-1.22)
High	1.05 (1.01-1.11)
Very high	1 [Reference]

Abbreviations: CHIP, Children’s Health Insurance Program; COI, Child Opportunity Index; OR, odds ratio.

^a^ Includes Native Hawaiian/Pacific Islander, American Indian, and Alaskan Native.

**Table 3.** Association of COI Level by Subdomain With Likelihood of Presenting With Complicated Appendicitis

COI	Adjusted OR (95% CI) of complicated appendicitis^a^
Education domain	
Very low	1.25 (1.18-1.32)
Low	1.22 (1.16-1.29)
Moderate	1.18 (1.12-1.25)
High	1.06 (1.00-1.11)
Very high	1 [Reference]
Health/environment domain	
Very low	1.23 (1.14-1.32)
Low	1.15 (1.08-1.24)
Moderate	1.08 (1.01-1.16)
High	1.05 (0.99-1.12)
Very high	1 [Reference]
Socioeconomic domain	
Very low	1.25 (1.18-1.33)
Low	1.19 (1.12-1.26)
Moderate	1.12 (1.06-1.18)
High	1.05 (0.99-1.11)
Very high	1 [Reference]

Abbreviations: COI, Child Opportunity Index; OR, odds ratio.

^a^ Adjusted for sex, age, race and ethnicity, insurance type, and complex chronic condition index.

For simple appendicitis cases, the proportion of patients who presented to the emergency department or were readmitted within 30 days from discharge ranged from 11.4% in very high COI to 13.5% in very low COI levels. For complicated appendicitis cases, the proportion of patients who presented to the emergency department or were readmitted within 30 days from discharge ranged from 20.8% in very high COI to 19.1% in very low COI levels. After covariate adjustment, there was no significant difference in the odds of unplanned postdischarge health care used based on COI for patients with either simple or complicated appendicitis (Table 4). For simple appendicitis cases, patients who were non-Hispanic Black (OR, 1.24; 95% CI, 1.07-1.43), Hispanic (OR, 1.21; 95% CI, 1.09-1.33), publicly insured (OR, 1.23; 95% CI, 1.13-1.33), and aged 0 to 4 years (OR, 1.58; 95% CI, 1.34-1.85) had increased odds of unplanned health care use. For patients with complicated appendicitis, those who were Hispanic (OR, 1.11; 95% CI, 1.02-1.22) and those aged 0 to 4 years (OR, 1.40; 95% CI, 1.24-1.57) had increased odds of unplanned health care use.

**Table 4.** Association of Overall COI Level With Unplanned Health Care Use After Discharge

Variable	ED visit and 30-d readmissions, OR (95% CI)	
	Simple appendicitis	Complicated appendicitis
COI level		
Very low	1.01 (0.90-1.14)	0.98 (0.88-1.09)
Low	0.91 (0.81-1.02)	0.91 (0.82-1.01)
Moderate	0.99 (0.89-1.11)	0.94 (0.85-1.05)
High	1.01 (0.91-1.13)	0.92 (0.83-1.02)
Very high	1 [Reference]	1 [Reference]
Sex		
Female	1.10 (1.03-1.18)	1.02 (0.96-1.09)
Male	1 [Reference]	1 [Reference]
Age, y		
0-4	1.58 (1.34-1.85)	1.40 (1.24-1.57)
5-9	1.06 (0.96-1.17)	1.12 (1.00-1.22)
10-14	0.97 (0.88-1.06)	1.06 (0.96-1.17)
≥15	1 [Reference]	1 [Reference]
Race/ethnicity		
Asian	0.89 (0.69-1.14)	1.16 (0.96-1.41)
Hispanic	1.21 (1.09-1.33)	1.11 (1.02-1.22)
Non-Hispanic, Black	1.24 (1.07-1.43)	1.13 (0.99-1.29)
Non-Hispanic, White	1 [Reference]	1 [Reference]
Other^a^	0.93 (0.82-1.05)	0.86 (0.77-0.96)
Complex chronic condition		
Yes	4.0 (3.56-4.44)	1.83 (1.63-2.06)
No	1 [Reference]	1 [Reference]
Insurance		
Public (Medicaid/CHIP)	1.23 (1.13-1.33)	1.03 (0.95-1.11)
Other	0.98 (0.85-1.13)	0.85 (0.76-0.97)
Private	1 [Reference]	1 [Reference]

Abbreviations: CHIP, Children’s Health Insurance Program; COI, Child Opportunity Index; OR, odds ratio.

^a^ Includes Native Hawaiian/Pacific Islander, American Indian, and Alaskan Native.

Limiting the definition of complicated appendicitis to *ICD-10-CM* code K35.2 in the sensitivity analysis resulted in 24.0% of the cases being complicated appendicitis, compared with 46.3% in the main analysis. Overall, differences in patient characteristics between simple and complicated appendicitis were consistent with the main analysis (eTable 2 in the Supplement). After adjustment, in comparison with very high COI, the odds of presenting with complicated appendicitis ranged from 1.07 (95% CI, 1.01-1.14) for high COI to 1.25 (95% CI, 1.17-1.33) for very low COI levels. Full regression results are presented in eTable 3 in the Supplement. The results for the secondary outcome were also consistent with the main analysis.

## Discussion

In this cohort study, we sought to examine the association between neighborhood SDOH, measured by the COI, and the odds of presenting with complicated appendicitis and using unplanned health care after discharge. Our findings suggest the importance of neighborhood-level SDOH and their association with access to care of a common pediatric surgical condition. Children from lower-COI neighborhoods had 5% to 28% increased odds of presenting with complicated appendicitis compared with those from the highest-COI neighborhoods, even after controlling for patient-level SDOH factors. There was no observed difference in postdischarge unplanned health care use based on COI level. These findings have important clinical and policy implications.

To our knowledge, our finding that lower COI level was associated with increased odds of presenting with complicated appendicitis has not been previously reported. This finding highlights the association of neighborhood SDOH factors with surgical access for this common pediatric surgical condition. This association is important because delays in the diagnosis and care of appendicitis increase the risk of complicated appendicitis, which is associated with increased rates of surgical complications, higher health care costs, longer lengths of stay, and increased rates of readmission.^4,13^ We also found that lower levels in all 3 COI subdomains (educational level, health/environment, and socioeconomic status) were associated with increased odds of presenting with complicated appendicitis and, thus, access to appendicitis care may benefit from strategically allocating resources and developing interventions in each domain.^21^ Although, to our knowledge, no studies have evaluated the association of neighborhood SDOH and pediatric surgical conditions, Carmichael et al^31^ reported an increased risk for emergent surgery, which is an independent negative risk factor for worse surgical outcomes, among adult patients from neighborhoods with a high social vulnerability index. Slater^32^ reported that adult patients undergoing surgery who are from neighborhoods of high social vulnerability have increased odds of extended length of stay, postoperative complications, and mortality and called for further efforts to integrate SDOH assessments into patient care and address community-level SDOH. In pediatric cardiac surgery, Anderson et al^7^ noted that children from low-income zip codes had an increased risk of mortality and intensive care resource use than children from high-income zip codes. Based on our findings, ensuring that patients have access to both diagnosis and definitive appendicitis care may require interventions that go beyond expanding insurance coverage.^33,34^

Our results did not note differences in unplanned postdischarge health care use for either the simple or the complicated appendicitis group based on COI level. Similar findings were reported in the nonsurgical literature, in which readmissions after pediatric asthma hospitalization were not significantly different based on COI level.^8^ This similarity suggests that perhaps once patients have established care, they are connected to a pediatric resource that can support families after discharge, regardless of the patient’s neighborhood. For example, parents can contact clinical staff with any questions or concerns regarding their child’s postoperative course, such as wound complications, bowel disturbances, fever, poor pain control, or reduced appetite or fluid intake. Timely access to clinical recommendations can help parents determine how to best care for their child and decide whether they require further evaluation in the clinic or emergency department. Health care professional triage over the telephone can therefore assist in reducing nonemergency visits to the emergency department and mitigate risk factors that may contribute to increased rates of postoperative readmission. Such findings may thus indicate that, to improve outcomes for children with appendicitis, policy makers should focus most of their efforts on developing interventions to ensure timely initial presentation.

Targeted interventions and investments may help to mitigate risk factors and reduce disparities in access to pediatric surgical care. The Affordable Care Act encouraged hospitals to spend more on community health improvements with community-based health intervention mandates.^35^ Meanwhile, accountable care organizations have an incentive to reduce morbidity of those they insure to reduce their overall costs.^36^ Public health departments, health insurance companies, local government, and regional health systems are thus increasingly interested in improving community health by implementing innovative approaches to enhance access to care. For example, some organizations have used telemedicine, mobile health care, and clinic expansion in underserved communities to increase access or have encouraged neighborhood development through creating safe, public spaces for exercise or increasing access to affordable, nutritious foods.^36^ Some organizations have also started reimbursing patients for transportation costs.^36^ Previous methods to address limited access to pediatric surgery include the use of telemedicine consultations for both patients and surgeons in remote or international regions with minimal ability to transfer the patient to a children’s hospital.^37,38,39^ In addition, regional networks for timely and effective management of patients who experience trauma have reported success in improving outcomes for children with reduced access to tertiary pediatric centers.^40,41^ Combined, these efforts may help to improve access to definitive care.

### Limitations

This study has limitations. First, the patient cohort was obtained from the PHIS database, which is limited to tertiary children’s hospitals and may include a higher proportion of complicated appendicitis cases compared with community hospitals; however, because the primary outcome was not the overall prevalence, we believe the odds of complicated appendicitis as a function of COI, which includes nearly all US census tracts, can still be evaluated. Furthermore, it has been established that complicated appendicitis is a prehospital occurrence and not dependent on the type of hospital the patient initially presents to for care or the time to appendectomy once antibiotics are initiated and the patient is transferred.^42^ In addition, unlike other databases, the PHIS includes data on both inpatient and outpatient cases, which is necessary for capturing both simple and complicated appendicitis cases. Second, tertiary children’s hospitals are primarily located in metropolitan centers; thus, this cohort may underrepresent patients living in rural communities. However, the PHIS identifies patients living in urban/suburban and rural communities based on zip code; patients were classified as rural in 8.2% of simple appendicitis cases and 10.3% of complicated appendicitis cases, which did not significantly differ; therefore, it is unlikely that any differences in rates of complicated appendicitis were owing to geographic distance. Third, the new *ICD-10-CM* coding system does not clearly distinguish between simple and complicated appendicitis; however, we attempted to follow the Agency for Healthcare Research and Quality definition of perforated appendicitis and completed a sensitivity analysis using code K35.2, a high-specificity indicator for perforated appendicitis, which was not significantly different from our primary analysis.^27,28,29,30^ Fourth, unplanned health care use may differ based on emergency department visits vs 30-day readmissions. The emergency department visits may be more prone to nonemergency health care use, whereas readmissions are more likely to represent true medical indications for admission after discharge. The PHIS does not allow for reliable breakdown of these 2 encounter types, and thus these factors needed to be combined into an unplanned health care use category. Fifth, owing to the large sample size of the cohort, small differences identified by statistical tests as significant may not be clinically meaningful; however, the increased rate of complicated appendicitis in patients who are of a racial or ethnic minority group, publicly insured, younger, and with a complex chronic condition is consistent with previous literature. Sixth, although complicated appendicitis is frequently used as an indirect marker of access to surgical care, patients may present with complicated appendicitis for other reasons, such as minimal or discordant symptoms, and thus may simply have a delayed presentation unrelated to access.

## Conclusions

In this cohort study, after accounting for patient-level SDOH, children from lower-COI neighborhoods had increased odds of presenting with complicated appendicitis, which can represent a delay in care frequently associated with increased complication risk, length of stay, cost, and readmission rate. These findings can inform future policy and public health interventions to increase community opportunity and promote equitable pediatric health outcomes across neighborhoods. Reducing appendicitis disparities may involve addressing neighborhood SDOH through neighborhood-focused investment, reallocation of community resources, and improved access to care in underserved communities. Furthermore, it may be useful for health professionals to continue patient screening for SDOH and refer patients to appropriate resources when unmet social needs are identified. The details provided by the COI can be incorporated into community needs assessments and inform targeted interventions by health systems, public health agencies, and insurance companies to improve child health and equity. Future research on the association of neighborhood SDOH and other pediatric surgical conditions and evaluation of the association of specific community interventions with pediatric health outcomes may be beneficial.

## Notes

Supplement. **eTable 1.** Agency for Healthcare Research and Quality *ICD-10* Codes Used to Identify Appendicitis Cases During the Study Period From October 1, 2015, Through September 30, 2018 **eTable 2.** Patient Characteristics for the Sensitivity Analysis, Where Complicated Appendicitis Cases Were Defined Only as Cases With *ICD-10* K35.2 **eTable 3.** Adjusted Odds Ratio (OR) of Presenting With Complicated Appendicitis Based on Each Covariate and Overall COI in the Sensitivity Analysis, Where Complicated Appendicitis Cases Were Defined Only as Cases With *ICD-10* K35.2 **eAppendix.** Additional Details on the Child Opportunity Index
