# Supplementary material for: Association Between Neighborhood-Level Social Determinants of Health and Access to Pediatric Appendicitis Care
Source: JAMA Netw Open. 2022 Feb 16;5(2):e2148865. doi: 10.1001/jamanetworkopen.2021.48865 (PMC8851303; doi:10.1001/jamanetworkopen.2021.48865)
Supplement: Supplement. — eTable 1. Agency for Healthcare Research and Quality ICD-10 Codes Used to Identify Appendicitis Cases During the Study Period From October 1, 2015, Through September 30, 2018 eTable 2. Patient Characteristics for the Sensitivity Analysis, Where Complicated Appendicitis Cases Were Defined Only as Cases With ICD-10 K35.2 eTable 3. Adjusted Odds Ratio (OR) of Presenting With Complicated Appendicitis Based on Each Covariate and Overall COI in the Sensitivity Analysis, Where Complicated Appendicitis Cases Were Defined Only as Cases With ICD-10 K35.2 eAppendix. Additional Details on the Child Opportunity Index [file jamanetwopen-e2148865-s001.pdf]

Supplementary Online Content

Bouchard ME, Kan K, Tian Y, et al. Association between neighborhood-level social determinants of health and access to pediatric appendicitis care. *JAMA Netw Open*. 2022;5(2):e2148865. doi:10.1001/jamanetworkopen.2021.48865

eTable 1. Agency for Healthcare Research and Quality *ICD-10* Codes Used to Identify Appendicitis Cases During the Study Period From October 1, 2015, Through September 30, 2018
eTable 2. Patient Characteristics for the Sensitivity Analysis, Where Complicated Appendicitis Cases Were Defined Only as Cases With *ICD-10* K35.2
eTable 3. Adjusted Odds Ratio (OR) of Presenting With Complicated Appendicitis Based on Each Covariate and Overall COI in the Sensitivity Analysis, Where Complicated Appendicitis Cases Were Defined Only as Cases With *ICD-10* K35.2
eAppendix. Additional Details on the Child Opportunity Index

This supplementary material has been provided by the authors to give readers additional information about their work.

© 2022 Bouchard ME et al. *JAMA Network Open.*

Code	Diagnosis
**K35.2**	Acute appendicitis with generalized peritonitis
**K35.3**	Acute appendicitis with localized peritonitis
**K35.80**	Unspecified acute appendicitis
**K35.89**	Other acute appendicitis
**K37**	Unspecified appendicitis

eTable 1: Agency for Healthcare Research and Quality *ICD-10* codes used to identify appendicitis cases during the study period from October 1, 2015, through September 30, 2018.

© 2022 Bouchard ME et al. *JAMA Network Open.*

	Total N	Simple N(%)	Complicated N(%)	P value
**N**	67489	51315 (76.0)	16174 (24.0)	
Patient Characteristics:				
**Age**				<0.001
Mean (SD)	10.5 (3.9)	10.8 (3.7)	9.5 (4.1)	
Median (Q1, Q3)	11 (8,14)	11 (8,14)	10 (6,13)	
Range	0, 18	0, 18	0, 18	
**Sex**				<0.001
Female	26929 (39.9)	20251 (39.5)	6678 (41.3)	
Male	40549 (60.1)	31056 (60.5)	9493 (58.7)	
Unknown	11 (0.02)	8 (0.0)	3 (0.0)	
**Race/Ethnicity**				<0.001
Asian	1699 (2.5)	1259 (2.5)	440 (2.7)	
Hispanic	24234 (35.9)	17658 (34.4)	6576 (40.7)	
Non-Hispanic Black	4447 (6.6)	3301 (6.4)	1146 (7.1)	
Non-Hispanic White	29234 (43.3)	23065 (44.9)	6169 (38.1)	
Other	7875 (11.7)	6032 (11.8)	1843 (11.4)	
**Insurance**				<0.001
Private	28991 (43.0)	22986 (44.8)	6005 (37.1)	
Public (Medicaid/CHIP)	32343 (47.9)	23691 (46.2)	8652 (53.5)	
Other	6155 (9.1)	4638 (9.0)	1517 (9.4)	
**Complex Chronic Condition**				0.001
Yes	3607 (5.3)	2659 (5.2)	948 (5.9)	
No	63882 (94.7)	48656 (94.8)	15226 (94.1)	
Patient Outcomes:				
**COI**				<0.001
Very Low	15488 (23.0)	11218 (21.9)	4270 (26.4)	
Low	13535 (20.1)	10055 (19.6)	3480 (21.5)	
Moderate	12668 (18.8)	9641 (18.8)	3027 (18.7)	
High	11487 (17.0)	8941 (17.4)	2546 (15.7)	
Very High	14311 (21.2)	11460 (22.3)	2851 (17.6)	
**ED Visit/ 30 day Readmission**				<0.001
Yes	10358 (15.4)	6948 (13.5)	3410 (21.1)	
No	57131 (84.7)	44367 (86.5)	12764 (78.9)	

eTable 2: Patient characteristics for the sensitivity analysis, where complicated appendicitis cases were defined only as cases with ICD-10 K35.2 (a high specificity indicator for complicated appendicitis). Age is reported in years. The “other” category in race/ethnicity includes Native Hawaiian Pacific Islander, American Indian and Alaskan Native**.** Abbreviations: n- number,

© 2022 Bouchard ME et al. *JAMA Network Open.*

CHIP- Children's Health Insurance Program, COI- Child Opportunity Index, ED- Emergency Department.

© 2022 Bouchard ME et al. *JAMA Network Open.*

Covariate	Adjusted complicated appendicitis OR (95% CI)
**Sex**	
Female	1.07 (1.03, 1.11)
Unknown	1.29 (0.34, 4.92)
Male	*Reference*
**Age**	
0-4	3.55 (3.29, 3.83)
5-9	1.68 (1.59, 1.78)
10-14	1.32 (1.25, 1.39)
≥15	*Reference*
**Race/Ethnicity**	
Asian	1.27 (1.13, 1.42)
Hispanic	1.21 (1.14, 1.27)
Non-Hispanic Black	1.15 (1.07, 1.25)
Other	1.02 (0.96, 1.09)
Non-Hispanic White	*Reference*
**Insurance**	
Public (Medicaid/CHIP)	1.13 (1.08, 1.18)
Other	1.08 (1.01, 1.17)
Private	*Reference*
**Complex Chronic Condition**	
Yes	1.11 (1.03, 1.20)
No	*Reference*
**COI Overall**	
Very Low	1.25 (1.17, 1.33)
Low	1.18 (1.11, 1.26)
Moderate	1.15 (1.08, 1.22)
High	1.07 (1.01, 1.14)
Very High	*Reference*

eTable 3: Adjusted odds ratio (OR) of presenting with complicated appendicitis based on each covariate and overall COI in the sensitivity analysis, where complicated appendicitis cases were defined only as cases with ICD-10 K35.2 (a high specificity indicator for complicated appendicitis). The COI OR was adjusted for the following covariates: sex, age, race/ethnicity, insurance type and complex chronic condition index. The “other” category in race/ethnicity

© 2022 Bouchard ME et al. *JAMA Network Open.*

includes Native Hawaiian Pacific Islander, American Indian and Alaskan Native**.** Abbreviations: CHIP- Children's Health Insurance Program, COI- Child Opportunity Index.

© 2022 Bouchard ME et al. *JAMA Network Open.*

eAppendix. Additional Details on the Child Opportunity Index

We used the Child Opportunity Index (COI) 2.0 Database to complete the study. The COI captures Zip code neighborhood opportunity level information and is available for nearly all U.S. Census tract neighborhoods using 2015 data compiled from public sources, including the Census Bureau, National Center for Health Statistics, Department of Education and the Environmental Protection Agency.[21] After a comprehensive, multidisciplinary literary review, the COI database compiled 29 independent indicators of neighborhood characteristics that are known to influence a child's health and development, including but not limited to, access to and quality of education, healthcare, green space, nutritious food, toxin-free environments, safety and socioeconomic resources.[21]

The COI indicators are further sub-divided into three themed subdomains: education, health/environment and social/economic.[21] The education domain includes early childhood education, elementary education, secondary and postsecondary education and educational and social resources. It also captures neighborhood-level education-related resources pertaining to children and adult educational attainment, which is known to influence children's health. The health and environment domain captures features that impact children's health in utero, childhood, and transition to adulthood; these factors can ultimately influence educational attainment and socioeconomic status. This domain primarily encapsulates neighborhood environmental factors to affect health behaviors and outcomes for children, such as access to food or green space and walkability. Finally, the social and economic domain incorporates economic opportunities and social resources, such as employment rates, length of commute to work, rates of single-headed households, public assistance rate, median household income and homeownership rates. All of these separate indicators are known to influence children's health, either directly or indirectly. [21]

The overall COI and subdomain scores are calculated from 1 to 100 and divided into nationally-normalized quintiles, from very low to very high opportunity level. The contribution of each indicator to overall score is weighted based on how strongly an indicator predicts long-term health outcomes.[21] The COI has been used to increase equity awareness, to evaluate how neighborhoods affect child health and to inform resource allocation, public health interventions, and policy development.[21,26]

© 2022 Bouchard ME et al. *JAMA Network Open.*
